# Genetic determinants of Nef-mediated CD4 and HLA class I down-regulation differences between HIV-1 subtypes B and C

**DOI:** 10.1186/s12985-015-0429-7

**Published:** 2015-11-25

**Authors:** Jaclyn K. Mann, Saleha Omarjee, Phumzile Khumalo, Thumbi Ndung’u

**Affiliations:** HIV Pathogenesis Programme, University of KwaZulu-Natal, 719 Umbilo Road, Durban, 4013 South Africa; KwaZulu-Natal Research Institute for Tuberculosis and HIV, University of KwaZulu-Natal, Durban, 4001 South Africa; Ragon Institute of MGH, MIT and Harvard University, Cambridge, MA 02139 USA; Max Planck Institute for Infection Biology, Chariteplatz, D-10117 Berlin, Germany

**Keywords:** HIV-1 Nef, HIV-1 subtypes, CD4 down-regulation, HLA-I down-regulation

## Abstract

**Background:**

HIV-1 subtype C Nef sequences have a significantly lower ability overall to down-regulate CD4 and HLA-I than subtype B Nef sequences. Here we investigated whether Nef amino acids differing in frequency between HIV-1 subtypes B and C explain lower CD4 and HLA-I down-regulation ability of subtype C.

**Findings:**

Subtype-specific mutations were introduced into representative subtype B and C Nef sequences and the CD4 and HLA-I down-regulation ability of these mutants was measured by flow cytometry in a CD4+ T cell line. Subtype C consensus 20I and subtype B consensus 20M reduced and increased HLA-I down-regulation respectively, and the S88G immune escape mutation (which is significantly more frequent in subtype C than subtype B) reduced CD4 and HLA-I down-regulation.

**Conclusions:**

Our data suggest that these subtype-specific differences may partly contribute to inter-subtype functional differences, and identification of an immune escape mutation – S88G – that impairs Nef function is of relevance to vaccine design.

The HIV-1 accessory protein Nef enhances viral replication and is critical for in vivo pathogenesis [1]. Infection with Nef-deleted HIV-1 strains is associated with long-term non-progression to AIDS, low plasma viral loads and maintenance of normal CD4 counts [2, 3]. Nef performs numerous activities that may contribute to its pathogenic effect, including the down-regulation of CD4 and HLA class I (HLA-I) molecules from the surface of infected cells. Down-regulation of CD4 promotes the budding of virions from infected cells thereby increasing viral replication, while down-regulation of HLA-I allows infected cells to evade recognition by cytotoxic T cells.

Recently, we reported that the CD4 and HLA-I down-regulation abilities of patient-derived Nef clones differed significantly between subtype A, B, C and D isolates; with subtype B clones displaying the highest CD4 and HLA-I down-regulation activity and subtype C clones displaying the lowest activity [4]. However, the mechanisms underlying these functional differences between subtypes remain unknown. A comparison of subtype B and C Nef sequences revealed that the consensus amino acids at codons 20, 35 and 59 differed between subtype B (encoding M, R and E, respectively) and subtype C (I, Q and Q, respectively) [4], and these three codons were reported to be involved in CD4 or HLA-I down-regulation by Nef [5–7]. Specifically, introduction of alanine or arginine at codon 20 resulted in significant loss of HLA-I down-regulation activity [7], mutation of residues 35 and 36 to alanine impaired CD4 down-regulation ability [5] and codon 59 is part of the CD4 binding site of Nef [5, 6]. Thus, these are candidate residues for mediating Nef functional differences between subtypes B and C. To identify additional Nef residues and naturally occurring amino acid variations that could potentially contribute to functional differences between subtypes, we examined statistical associations (not yet confirmed experimentally) between amino acid polymorphisms and altered down-regulation ability by patient-derived Nef sequences [4], and then compared the frequency of these amino acids between subtypes. Differences in the frequency of amino acids at Nef codons 8, 9, 11, 14 and 88 between subtypes coincided with the functional hierarchy observed among viral subtypes [4]. Specifically, consensus amino acids 8S, 9S, 11 V and 14P (which were the consensus residues for all four subtypes) were statistically associated with lower HLA-I down-regulation ability and were significantly less frequent in subtype B, which displayed the highest HLA-I down-regulation ability. Furthermore, the mutation S88G was statistically associated with lower CD4 down-regulation and was more frequent in subtype C, which displayed the lowest CD4 down-regulation ability. Therefore, we hypothesised that residues 8–14 and 88 might also contribute to observed functional differences between subtypes B and C.

In the present study, we directly assessed the effect of subtype-specific mutations at these Nef codons of interest on CD4 and HLA-I down-regulation ability in order to investigate whether these sequence variations contribute to the inter-subtype differences in Nef function. The list of mutations tested is shown in Table 1. Mutations were introduced into a subtype B Nef sequence, derived from the laboratory-adapted HIV-1 NL4-3 reference strain (which was first mutated to the subtype B consensus residue valine at codon 11 since the wild-type contains isoleucine at this position), as well as a patient-derived subtype C Nef sequence (SK68, called C13 in our prior study; Genbank accession KC906737), the Nef sequence from those described in [4] that had both the consensus residues at the codons of interest and the highest similarity to the consensus C sequence. In addition, mutations at codons 8–14 naturally present in a patient-derived subtype B Nef sequence (B47; Genbank accession KC906941) were reverted to consensus B residues to assess their impact on Nef function. Figure 1 shows the first 100 residues of Nef sequences NL4-3, SK68 and B47 relative to consensus B and C sequences [4] and highlights sites of mutation. Mutations were introduced into the Nef sequences by site-directed mutagenesis [8] using the QuikChange II XL Site-Directed Mutagenesis kit (Stratagene, USA), according to the manufacturer’s instructions.

**Table 1:** List of mutations introduced into NL4-3, SK68 and B47 Nef sequences

NL4-3	SK68	B47
S8R	S8R	
S9R	S9R	
V10-/V11-	I10-/V11-	
	S9-/I10-/V11-/G12-	
P14S	P14S	
S8L/V10-/V11-/P14A/A15S	S8L/I10-/V11-/P14A/A15S	
S8R/V11G/P14S	S8R/V11G/P14S	R8S/G11V/S14P
M20I	I20M	
R35Q	Q35R	
E59Q	Q59E	
S88G	S88G	
S88G/E98D	S88G/E98D	
E98D	E98D

**Fig. 1:**
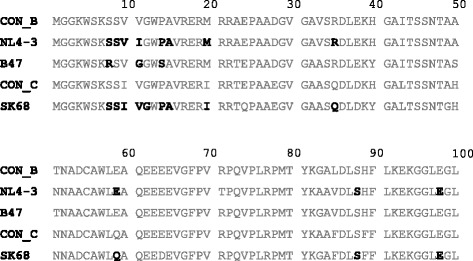
Subtype B and C Nef sequences and sites of mutation. The first 100 codons of the subtype B Nef sequences (NL4-3 and B47) and the subtype C Nef sequence (SK68) into which mutations were introduced are shown relative to the consensus B (Con_B) and consensus C (Con_C) Nef sequences. Sequences were aligned to HXB2 (and the subtype C specific insertion in the _62_EEEE_65_ motif with respect to HXB2 was stripped out). Codons at which mutations were introduced are shown in bold. It should be noted that, prior to commencing experiments, the NL4-3 Nef sequence was mutated to the subtype B consensus residue valine at codon 11 since the wild-type contains isoleucine

The CD4 and HLA-I down-regulation activities of the mutant Nef sequences were measured as previously described [4]. Briefly, the Nef sequences were cloned into a green fluorescent protein (GFP) reporter expression plasmid. Mutant Nef clones were then electroporated into a CEM-derived CD4 T cell line engineered to express high levels of HLA-A*02. Cell surface levels of CD4 and HLA-A*02 were measured using fluorescently-labelled antibodies specific for these molecules and flow cytometry. The median fluorescent intensity (MFI) of CD4 or HLA-A*02 in cells successfully transfected with Nef clones (GFP-expressing cells) was first expressed relative to the MFI of the SF2 Nef positive control and the empty plasmid negative control included in each experiment by the following equation: (negative control – Nef clone)/(negative control – positive control), where a value of 0 indicates no down-regulation activity and a value of 1 indicates down-regulation activity equivalent to SF2. Then, for comparison of mutant and wild-type clones, the CD4 and HLA down-regulation values for all mutant Nef clones were expressed as a percentage of the relevant wild-type (NL4-3, SK68 or B47) such that the wild-type represented 100 % down-regulation activity. Experiments were performed at least in triplicate and results averaged. For mutants that differed by more than 10 % in function from the wild-type, T tests with a p value cut-off of 0.05 were performed to test for significant difference from the wild-type.

Representative flow cytometry plots of the assay controls – empty plasmid negative control (showing no down-regulation activity) and SF2 Nef positive control (showing efficient down-regulation activity) – are shown in Fig. 2. We previously observed that differences in Nef-mediated CD4 and HLA-I down-regulation between consensus B and C Nef sequences were more apparent in cells expressing lower levels of Nef protein [4]. Since CD4 down-regulation function is highly conserved (our previous measurements for this function showed a very narrow spread of values across different Nef clones) we analysed CD4 down-regulation activity based on all GFP-expressing cells as well as the lower third of GFP-expressing cells only. Results were consistent for both gating strategies but differences in CD4 down-regulation between mutants were slightly greater (and therefore more clearly observed) in cells expressing lower levels of the Nef protein, therefore these results are presented here. Replicate measurements for both Nef functions were in excellent agreement (Pearson’s correlation *r* ≥ 0.94 and *p* < 0.0001) as shown previously using this method [4].

**Fig. 2:**
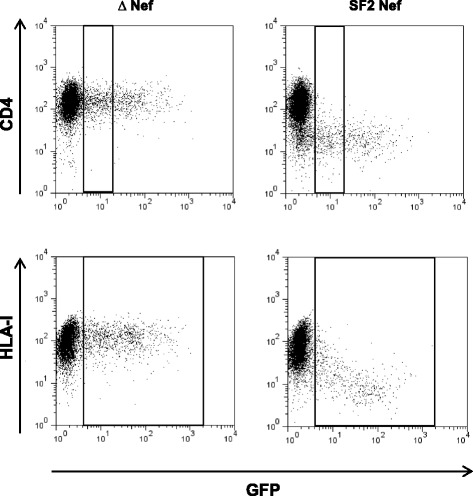
Representative flow cytometry plots of assay controls and Nef expression. Plots show cell-surface expression of CD4 or HLA-I (y axis) in cells transfected with empty plasmid (∆ Nef, negative control) and plasmid expressing SF2 Nef (positive control). Green fluorescent protein (GFP) expression is shown on the x axis and is an indicator of cells successfully transfected with plasmids. CD4 expression was measured in the lower third of GFP-expressing cells and HLA-I expression was measured in all GFP-expressing cells (gates shown on plots)

At the codons that were previously reported to be involved in CD4/HLA-I down-regulation and where the consensus amino acids differed between subtypes B and C, namely codons 20, 35 and 59, the subtype B Nef (NL4-3) was mutated to the subtype C consensus residue (20I, 35Q, and 59Q) and the subtype C Nef (SK68) was mutated to the subtype B consensus residue (20 M, 35R, and 59E). It was hypothesised that the subtype C consensus residues would decrease the down-regulation ability of NL4-3 and that the subtype B consensus residues would increase the down-regulation ability of SK68. As predicted, NL4-3 M20I displayed significantly reduced HLA-I down-regulation ability (to 69 % of wild-type levels, *p* < 0.0001, Fig. 3a) while HLA-I down-regulation ability was significantly increased in SK68 by the I20M mutation (to 118 % of wild-type levels, *p* = 0.0087, Fig. 3b), indicating that subtype-specific differences at codon 20 may contribute to the lower HLA-I down-regulation ability of subtype C Nefs relative to subtype B Nefs. Consistent with this, we recently reported that I20M was statistically associated with higher HLA-I down-regulation in 298 subtype C patient-derived Nef sequences [9]. However, a recent report suggested that M20I modestly enhanced the HLA-I down-regulation function of the subtype B Nef sequence SF2 [10], suggesting that other polymorphisms in Nef can modulate the effect of codon 20. Additional studies will be necessary to elucidate this. Nevertheless, our results support the conclusion that subtype-specific residues at codon 20 can affect HLA-I down-regulation ability and that this may partly explain inter-subtype differences in this Nef function. On the other hand, we observed that subtype-specific mutations at codons 35 and 59 (in regions previously described to be involved in CD4 down-regulation [5, 6]) did not appreciably affect CD4 down-regulation ability of NL4-3 (Fig. 3c) or SK68 (Fig. 3d). Thus, we found no evidence that subtype-specific differences at these codons contribute to differences in this Nef function between subtypes B and C.

**Fig. 3:**
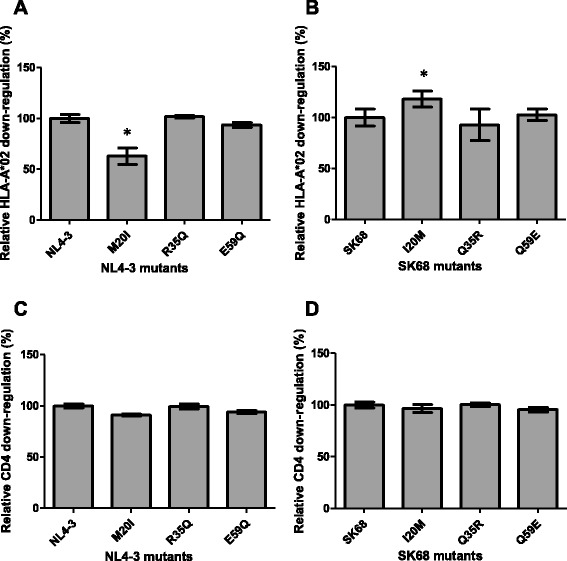
CD4 and HLA-I down-regulation ability of Nefs mutated to subtype B and C consensus residues. The HLA-I down-regulation ability of the subtype B NL4-3 Nef mutated to subtype C consensus residues 20I, 35Q and 59Q (panel **a**) and the subtype C SK68 Nef mutated to subtype B consensus residues 20 M, 35R and 59E (panel **b**) is shown. Similarly the CD4 down-regulation ability of these mutants is shown in panels **c** and **d**. Down-regulation activity is expressed relative to the respective wild-type protein (NL4-3 or SK68), which represents 100 %. The CD4 and HLA-I down-regulation ability expressed relative to SF2 was 97 and 99 % respectively for NL4-3, and 89 and 69 % respectively for SK68. Bars represent the mean of at least 3 replicates, and error bars represent standard deviations from the means. Asterisks indicate mutant clones that were > 10 % different in function from the wild-type and were significantly different from the wild-type as indicated by the Student’s T test (*p* < 0.05)

The S88G mutation was statistically associated with reduced CD4 down-regulation ability in patient-derived subtype C Nef sequences in our prior study [4]. To directly test whether the S88G mutation decreases CD4 down-regulation ability, this mutation was introduced into both NL4-3 and SK68. Furthermore, we found that the S88G mutation seldom occurred in the absence of the co-varying mutation E98D (considering subtype C sequences reported in [4], 81 % of sequences encoding S88G also harboured E98D), therefore the functional effect of these mutations alone as well as in combination was tested. Consistent with our hypothesis based on statistical associations, we observed that the S88G mutation significantly reduced CD4 down-regulation ability of NL4-3 (to 79 % of wild-type levels, *p* < 0.0001, Fig. 4a) and SK68 (to 53 % of wild-type levels, *p* < 0.0001, Fig. 4b). Interestingly, S88G was also observed to negatively affect HLA-I down-regulation activity of both Nefs (*p* < 0.0001 for both, Fig. 4c and d). As expected, when S88G was present together with E98D down-regulation activity was higher than when S88G was present alone, indicating that E98D functions as a compensatory mutation. However, E98D did not fully restore CD4 or HLA-I down-regulation function to wild-type levels in SK68 Nef sequences harbouring the S88G mutation (*p* = 0.0002 and *p* = 0.0004, respectively), and the NL4-3 S88G/E98D double mutant remained impaired for HLA-I down-regulation activity (*p* < 0.0001), indicating that E98D only partially compensated for the loss of function associated with S88G. Since the S88G mutation occurs more frequently in subtype C Nef sequences than subtype B Nef sequences, these results suggest that it may contribute to the overall lower CD4 down-regulation and HLA-I down-regulation activity observed for subtype C.

**Fig. 4:**
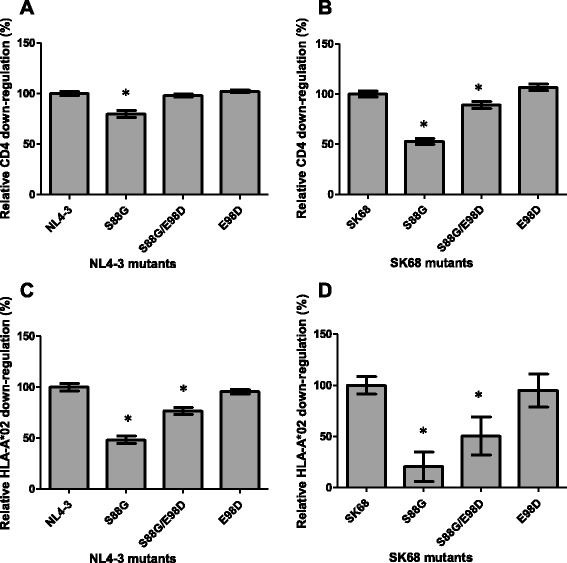
CD4 and HLA-I down-regulation ability of Nefs encoding escape and co-varying mutations. The CD4 down-regulation ability of the subtype B NL4-3 Nef (panel **a**) and the subtype C SK68 Nef (panel **b**) encoding the S88G mutation as well as S88G accompanied by the co-varying E98D mutation is shown. Similarly the HLA-I down-regulation ability of these mutants is shown in panels **c** and **d**. Down-regulation activity is expressed relative to the respective wild-type protein (NL4-3 or SK68), which represents 100 %. The CD4 and HLA-I down-regulation ability expressed relative to SF2 was 97 and 99 % respectively for NL4-3, and 89 and 69 % respectively for SK68. Bars represent the mean of at least 3 replicates, and error bars represent standard deviations from the means. Asterisks indicate mutant clones that were > 10 % different in function from the wild-type and were significantly different from the wild-type as indicated by the Student’s T test (*p* < 0.05)

Interestingly, S88G is an escape variant selected by CD8+ T cell responses targeting the HLA-B*58:01-restricted KF9 epitope [11]. Whether selection of Nef S88G contributes to the protective effect seen for HLA-B*58:01 should be examined in future studies. Regardless, the identification of such viral escape mutations with functional costs is informative for an attenuation-based HIV vaccine strategy [12]. For such a vaccine strategy, targeting multiple regions of the virus where escape has a fitness/functional cost may result in delayed immune escape (due to the consequent fitness cost) or viral attenuation following the development of escape mutations and consequently an attenuated disease course.

The most common mutations at codons 8, 9, 11 and 14 were introduced into the subtype B Nef (NL4-3) and subtype C Nef (SK68) in order to test the hypothesis that mutations at these codons increase HLA-I down-regulation activity. Since a deletion was the most common mutation observed at codon 11 and this was frequently accompanied by a deletion at codon 10 in subtype B and C, as well as deletions at codons 9, 10 and 12 in subtype C, we also studied these combinations. Furthermore, we observed that mutations at codons 8–14 co-varied and the patterns of mutations were extremely diverse, therefore we introduced a pattern observed twice in patient-derived subtype B Nef sequences – S8L/V10-/V11-/P14A. In both cases where this pattern occurred there were additional mutations in close proximity; we selected the S8L/V10-/V11-/P14A/A15S mutation combination.

We found that mutations at codons 8–14 did not increase HLA-I down-regulation activity of either NL4-3 or SK68 (Fig. 5a and b), and did not appreciably affect CD4 down-regulation (Fig. 5c and d). Since wild-type NL4-3 down-regulated HLA-I very well (approximately equivalent to SF2 Nef in our assays), it was perhaps not surprising that we did not observe a positive effect of mutations at codons 8–14 on the HLA-I down-regulation activity of NL4-3. However, we also reverted a combination of common mutations at codons 8, 11, 14 – S8R/V11G/P14S – to the consensus residues in a patient-derived subtype B sequence (B47) in order to test whether this would decrease HLA-I down-regulation activity and we did not observe this effect (Fig. 5a). With the exception of SK68 S9-/I10-/V11-/G12-, all mutations at codons 8-14 moderately decreased HLA-I down-regulation activity of SK68, and this was significant for the 9R, 14S, S8L/I10-/V11-/P14A/A15S and S8R/V11G/P14S mutants (*p* = 0.034, *p* = 0.047, *p* = 0.0029, and *p* = 0.0051, respectively, Fig. 5b). However, it should be noted that except for S9-/I10-/V11-/G12- the mutation combinations tested were not observed in subtype C Nef sequences, which may explain this finding. Overall, we found no evidence that mutations at codons 8–14 enhance HLA-I down-regulation activity and thus contribute to differences in HLA-I down-regulation activity between subtypes B and C. Yet, two independent studies have shown statistical associations between mutations within this region and increased HLA-I down-regulation activity [4, 13]. In addition, deletions in this region were previously reported to associate with weaker selection pressure across Nef indicating that they are involved in immune escape [14], which is consistent with our hypothesis that deletions in this region enhance HLA-I down-regulation. It is possible that we did not adequately capture the diverse patterns of mutation in this region, and further experiments may be required to clarify the role of codons 8–14 in HLA-I down-regulation.

**Fig. 5:**
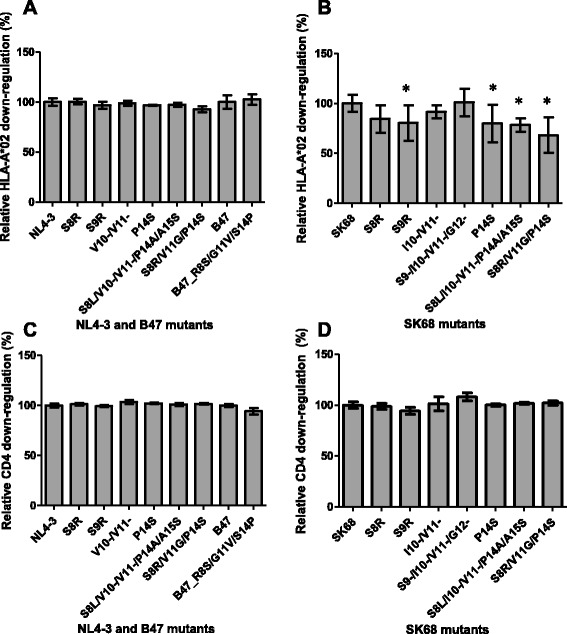
CD4 and HLA-I down-regulation ability of Nefs encoding mutations at codons 8-14. The HLA-I down-regulation ability of the subtype B NL4-3 Nef (panel **a**) and the subtype C SK68 Nef (panel **b**) encoding mutations and mutation combinations at codons 8–14 is shown. In addition, panel **a** shows the HLA-I down-regulation ability of the subtype B B47 Nef which naturally encoded the 8R/11G/14S mutation combination and was subsequently mutated to the consensus residues 8S, 11 V and 14P. Similarly the CD4 down-regulation ability of these mutants is shown in panels **c** and **d**. Down-regulation activity is expressed relative to the respective wild-type protein (NL4-3, SK68 or B47), which represents 100 %. The CD4 and HLA-I down-regulation ability expressed relative to SF2 was 97 and 99 % respectively for NL4-3, 89 and 69 % respectively for SK68, and 94 and 92 % respectively for B47. Bars represent the mean of at least 3 replicates, and error bars represent standard deviations from the means. Asterisks indicate mutant clones that were > 10 % different in function from the wild-type and were significantly different from the wild-type as indicated by the Student’s T test (*p* < 0.05)

We reasoned that Nef expression levels may influence CD4 and HLA-I down-regulation functions and therefore performed Western blot analysis, as described previously [4], to explore the impact of mutations on protein expression levels. Whereas the expression of NL4-3-derived Nef proteins were quantified, SK68-derived Nef proteins were undetectable by the antibodies used. NL4-3-derived Nef mutants M20I, S88G and S88G/E98D were the only mutants that displayed lower expression than the wild-type (43, 29 and 57 % of wild-type levels, respectively; data not shown). Interestingly, these were the same mutants that showed lower HLA-I down-regulation activity (however only S88G had significantly lower CD4 down-regulation activity), suggesting that reduced protein expression may be a mechanism contributing to diminished HLA-I down-regulation and that Nef expression may influence HLA-I down-regulation more than CD4 down-regulation.

In summary, we demonstrate here that subtype-specific differences at Nef codons 20 and 88 significantly affect this protein’s down-regulation activity. The subtype C consensus residue 20I reduced HLA-I down-regulation activity in subtype B and C Nef sequences and S88G, which is observed more frequently in subtype C Nef sequences compared to subtype B sequences, appreciably reduced CD4 and HLA-I down-regulation activity in Nef clones from both subtypes. Furthermore, we found that E98D was able to partially compensate for S88G. Our findings suggest that subtype-specific differences at these codons contribute to lower down-regulation activity of subtype C Nef clones compared to those from subtype B. Furthermore, the identification of a significant functional cost associated with the escape mutation S88G may have implications for vaccine strategies that aim to focus the immune response on particularly vulnerable regions of the virus where escape mutations impair protein function.

## Abbreviations

CD4: cluster of differentiation 4
GFP: green fluorescent protein
HIV-1: human immunodeficiency virus type 1
HLA: human leukocyte antigen
Nef: negative regulation factor
